# Coccidioidomycosis in a reference center in Northeast Brazil: clinical/epidemiological profile and most common radiological findings

**DOI:** 10.1590/0037-8682-0249-2020

**Published:** 2020-10-21

**Authors:** José Leonardo da Silveira Morais, Maria Clara Machado Borges, Letícia Maria Mota Braga Cavalcante, Paula Vitória Pereira Motoyama, Mariana Pitombeira Libório, Lara Gurgel Fernandes Távora

**Affiliations:** 1 Universidade de Fortaleza, Faculdade de Medicina, Fortaleza, CE, Brasil.

**Keywords:** Coccidioidomycosis, Coccidioides, Mycoses

## Abstract

**INTRODUCTION:**

Coccidioidomycosis, a disease caused by *Coccidioides immitis* or *Coccidioides posadasii*, is endemic in arid climatic regions in Northeast Brazil. Its prevalence is higher among young adult males living in rural areas. Existing literature about this disease in Ceará, a Northeast Brazilian state, are scarce. Here, we aimed to outline the clinical and epidemiological profiles, radiological patterns, and therapeutic responses of patients with coccidioidomycosis in a reference center in Ceará, Brazil.

**METHODS:**

This is a descriptive study with quantitative analysis. Patients who underwent medical follow-up in São José Hospital of Infectious Diseases and received confirmed mycological diagnosis of coccidioidomycosis between January, 2007 and December 2017 were included. Epidemiological, clinical, radiological, and therapeutic response data were collected from medical charts.

**RESULTS:**

Thirty patients were included. The patients were males with median age of 30 years, and 73% were considered to have high-risk exposure to *Coccidioides* owing to professional activities. Cough (96.7%), dyspnea (63.3%), fever (86.7%), and pleuritic pain (60%) were the most prevalent clinical manifestations. Interstitial pattern (91.3%) was the most frequent pulmonary radiological finding. Fluconazole, amphotericin B, and itraconazole were administered for treatment (in 82.1%, 42.8%, and 21.4% of cases, respectively). A favorable outcome was observed in 83.8% of patients.

**CONCLUSIONS:**

Coccidioidomycosis was more prevalent in the central and southern regions of the State of Ceará. Understanding the local epidemiology and clinical manifestations of the disease, in addition to the pulmonary radiologic findings, may aid the early detection of coccidioidomycosis and facilitate early diagnosis.

## INTRODUCTION

Coccidioidomycosis is a fungal infection with a favorable outcome in most cases. Its etiological agents are *Coccidioides immitis* or *Coccidioides posadasii*, which infect humans through inhalation of infective conidia. These fungal structures are naturally found in soil in specific geographical areas, and are transmitted during activities that involve soil handling^1^.

Data regarding the real incidence of coccidioidomycosis in Brazil are currently unavailable as it is not compulsory notification disease^2^. Despite this, coccidioidomycosis is considered an endemic in several areas in Northeast Brazil. Its persistence is associated with the arid climate in the region. It has a higher prevalence among young male adults (aged 20 to 50 years) living in rural areas. Certain activities, such as agriculture, construction, and hunting, especially armadillo hunting, which are common in Northeast Brazil, increase the risk of infection^3^. This is owing to a higher exposure of these individuals to fungal microniche disturbances in soil, with a higher probability of conidia inhalation and infection^4^.

In 1999, a report described eleven confirmed and three possibly autochthonous cases of coccidioidomycosis in four Northeast Brazilian states: Bahia, Ceará, Piauí, and Maranhão^5^. Between 1995 and 2007, 19 cases were reported in Ceará State in Northeast Brazil^6^ ^-^ ^8^. Since then, data have not been added adequately, which makes it difficult to understand the clinical and epidemiological aspects of coccidioidomycosis in this population. 

In this study, we aimed to outline the clinical and epidemiological profiles, radiological patterns, and therapeutic responses of patients with coccidioidomycosis in a reference center in Ceará, Brazil.

## METHODS

This is a cross-sectional descriptive study involving quantitative analysis. Patients who underwent medical follow-up in São José Hospital of Infectious Diseases and received confirmed mycological diagnosis of coccidioidomycosis, between January, 2007 and December, 2017 were included. 

A confirmed mycological diagnosis entailed the direct visualization of mature spherules, a positive biological specimen culture, or histopathological exam result in the medical chart. Epidemiological (gender, age, municipality, profession, education level, risk exposure), clinical (comorbidities, clinical manifestation of the disease, most prevalent signs and symptoms, and complications), radiological, and therapeutic response data were collected from the medical charts and radiological image files of the patients. Statistical analysis was performed using Statistical Package for the IBM Social Sciences (SPSS 16.0) software.

The study was approved by the São José Hospital Ethics and Research Committee (protocol n^0^ 2.405.644). 

## RESULTS

Thirty patients were included. The patients were male, with median age of 30 years (standard deviation: 13.1). 

The sources of infection were identified in 80% of the cases. They were related to armadillo hunting and artesian well digger activities (23 cases and 1 case, respectively). Farm work was considered the most probable source of infection in four patients. 

Only two patients exhibited comorbidities: one had systemic arterial hypertension and another reported the use of immunosuppressive medication. 

**FIGURE 1:**
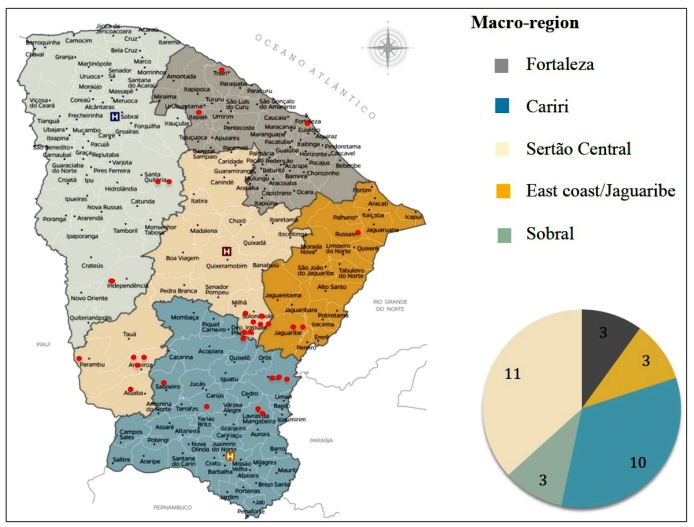
Incidence of coccidioidomycosis in a reference center in Ceará, Northeast Brazil - geographical distribution based on macroregions, January, 2007 - December, 2017. **Macroregions:** Fortaleza; Cariri; Sertão Central; East Coast/Jaguaribe; Sobral. **In red:** Cases.

Municipalities were categorized based on one of the five macroregions of the state, depending on the location of the hometown of the patients: Fortaleza, Sobral, Cariri, Sertão Central, and East Coast/Jaguaribe. The majority of cases were from Cariri and Sertão Central macroregions, and the city of Solonopole showed the highest prevalence, with six cases reported (Figure 1).

Most patients reported experiencing the first episode of coccidioidomycosis. Only one patient reported a second episode occurrence according to medical records. Based on the information from the medical charts, it was not possible to elucidate whether the second episode was secondary to reinfection or a disease relapse. The median time interval between the onset of symptoms and the first medical evaluation at São José Hospital was 20 days (minimum 8, maximum 365).

**FIGURE 2:**
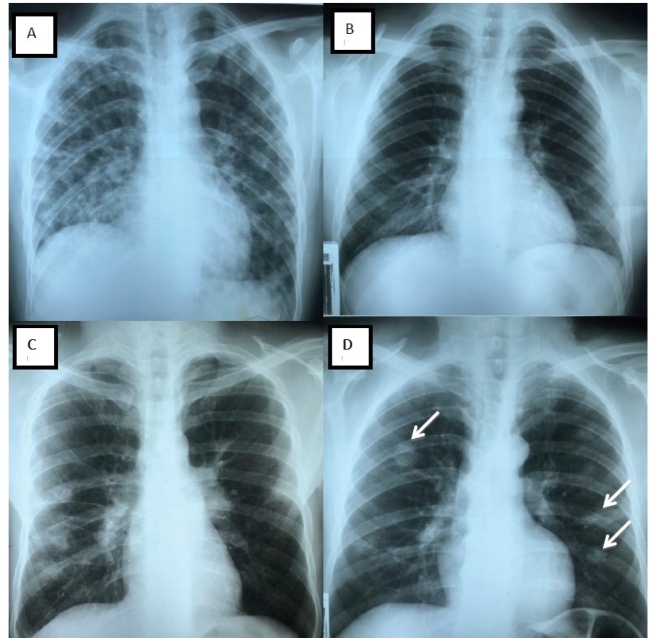
Patterns observed in the chest X-ray of patients with Coccidioidomycosis in a reference center in Ceará, Northeast Brazil. **A:** diffuse reticulonodular pattern. **B:** reticular pattern in left lower lung. **C:** peripheral nodular right lung lesions, presence of hilar lymphadenopathy. **D:** multiple nodular lesions (arrows).

The most prevalent clinical manifestations were cough (96.7%), dyspnea (63.3%), fever (86.7%), and pleutitic pain (60%) (Table 1). Considering these findings, chest radiographs at the time of admission were essential for an accurate initial evaluation of suspected cases, and were available for 96.7% of patients. Only chest X-ray reports certified by a radiology specialist were included in the present study. The most prevalent pattern was interstitial (91.3%), with isolated nodular or reticular involvement being reported equally (34.7%). It was not possible to access official radiological reports in six cases (Table 2; Figure 2).

**TABLE 1:** Clinical manifestations in patients with coccidioidomycosis in a reference center in Ceará, Northeast Brazil, January, 2007 - December, 2017.

Clinical manifestations	Number (%)
Cough	29 (96.7)
Fever	26 (86.7)
Dyspnea	19 (63.3)
Pleuritic/thoracic pain	18 (60)
Expectoration	13 (43.3)
Weight loss	13 (42.3)
Fatigue	8 (26.7)
Chills	4 (13.3)
Hemoptysis	2 (6.7)
Total	30 (100)

**TABLE 2:** Most prevalent radiologic findings in chest radiographs of patients with coccidioidomycosis in a reference center in Ceará, Northeast Brazil - January, 2007 - December, 2017.

Radiological patterns	Number (%)
Interstitial	
Reticular	8 (34.7)
Nodular	8 (34.7)
Reticulonodular	5 (21.7)
Cavitation	1 (3.4)
Alveolar consolidation	4 (17.3)
Other radiological findings	Number (%)
Pleural effusion	4 (17.3)
Lymphadenopathy	4 (17.3)
**Total**	**23**

In most cases, diagnosis was confirmed based on microbiological tests (direct mycological examination and culture); sputum being the most frequently used biological specimen (85.7%), followed by bronchoalveolar lavage (28.5%). More than one biological specimen was collected from four patients (13.3%) for diagnostic investigation. A positive culture result was reported in two patients. Only one patient was diagnosed with transbronchial biopsy. 

Twenty-eight patients were treated at São José Hospital, and two were transferred before diagnostic conclusion and initiation of specific therapy. Fluconazole, amphotericin B, and itraconazole were the most frequently prescribed drugs (in 82.1%, 42.8%, and 21.4% cases, respectively). Eleven (39.2%) patients underwent sequential therapy, with the administration of amphotericin B, followed by either fluconazole or itraconazole, prescribed in 90% of the cases. Treatment with fluconazole alone was prescribed for 12 patients (42.8%). 

With respect to patient outcomes, 83.8% of them were considered cured by the assistant physician. In four patients (13.3%), the outcomes at follow-up were not recorded because they were transferred to other medical centers. Only one death was reported. 

## DISCUSSION

In the present study, the patients were young males (median age, 30 years), living in rural areas. The data from this study corroborate those from other published studies that indicated that this disease was predominant among males with age ranging between 19 and 43 years^3^ ^,^ ^9^.

The most common type of activity for exposure was armadillo hunting. Similar results were published by Deus Filho *et al* (2010)^4^, where 96.4% of cases were related to armadillo hunting, and only one case was related to well digging. In a coccidioidomycosis case review for the State of Ceará from 1995-2007, Cordeiro *et al* ^3^ reported that armadillo hunting was the most important source of exposure^3^. Other risk factors for *Coccidioides* infections were described as well: construction work, farm work, and work in archaeological fields^10^. In our case series, the assistant physicians considered farm work the probable source of infection in four patients. 

The highest number of cases in this study were reported in Sertão Central and Cariri macroregions. Arid and semi-arid areas with sandy alkaline soil and extreme temperatures play an important role in the environmental colonization of *Coccidioides* ^5^ ^,^ ^11^ ^,^ ^12^. This leads to arthroconidia formation and their subsequent release in the air, which allows their inhalation by a susceptible host^13^. According to data from Research and Economic Strategy Institute of Ceará (*Instituto de pesquisa e estratégia econômica do Ceará* - IPECE), the State of Ceará exhibits several of the climate and soil characteristics discussed above, such as dry weather, low rainfall, soil with alkaline pH, and high salinity. This report indicates that Sertão Central and North Cariri regions have semi-arid hot tropical climate, and share multiple important climatic characteristics with areas with endemic coccidioidomycosis; this might explain the higher disease prevalence in these regions^14^.

In the present study, patients presumably presented with acute pulmonary coccidioidomycosis, and the most common clinical manifestations included cough, dyspnea, and pleuritic pain. According to data, approximately 60% of coccidioidomycosis cases are symptomatic^15^. In the acute phase, the clinical presentation of coccidioidomycosis may resemble that of an acute bacterial or viral infection, based on the predominant symptoms of the disease (fever, cough, and pleuritic chest pain)^3^ ^,^ ^9^ ^,^ ^16^. In endemic regions, acute pulmonary coccididoidomycosis accounts for 29% of community-acquired penumonia^17^.

Chest radiography was performed at admission for 29 patients. Interstitial patterns, specially nodular and reticular patterns, were the most common findings. Imaging tests play an important role in the differential diagnosis of coccidioidomycosis and evaluation of pulmonary involvement extension. In acute phases of the disease, the most common findings in chest radiographs are segmental or parenchymal lobar consolidation (75% of cases)^15^ ^,^ ^18^. However, the radiological presentations may vary, ranging from alveolar or reticulonodular infiltrates with or without pleural effusion to multiple cavities. Complications, such as empyema and bronchopleural fistulas, may also be found^6^. In this series of cases, a nodular pattern was reported in 27.5% of the cases. In a descriptive study on 15 patients with confirmed acute pulmonary coccidioidomycosis, Capone *et al* reported that the most frequent radiological finding was nodular pattern, with multiple nodules detected in 86.7% of patients, predominantly in the inferior lobes^19^.

The presence of cavitation has also been described in literature, although at a lower frequency, in less than 10% of the cases^20^ ^,^ ^21^. It is generally best visualized by computerized tomography, which can also help detect mediastinal and hilar lymph node enlargement in a large number of cases. In this study, cavitation was less frequent as well (3.4% of cases).

Pleural effusion occurs in approximately 15%-20% of cases and is usually characterized by pulmonary parenchymal involvement^18^. In our case series, 13.8% of the patients presented with pleural effusion on admission. These patients also exhibited parenchymal pulmonary involvement.

The diagnosis of coccidioidomycosis can be confirmed using direct mycological examination, biological specimen culture, and serological methods^13^. Although fungus detection in pathological specimens or cultures is the gold standard for establishing diagnosis, serological testing and direct mycological visualization are most widely used^22^.

 Direct mycological examination of respiratory specimens is frequently performed owing to its high sensitivity and low cost^21^. Immunological serological assays have high specificity, and can detect IgM in the initial course of the disease (until 7 days from the onset of symptoms) and IgG during a later period (after 2-3 weeks). However, the high cost of the reagents can be attributed for the low popularity of this technique for the diagnosis of coccidioidomycosis. Certain authors have also discussed the diagnostic challenges of using serologic testing in the early course of the disease. The tests may yield negative results in the initial phase, which can potentially lead to misdiagnosis^22^ ^,^ ^23^. In the current study, direct mycological examination of respiratory specimens was the most frequently used method for confirming the diagnosis. We believe that the higher costs of serological tests limit their use at our center.

In this study, we observed that only 39.2% of patients received sequential treatment with amphotericin B followed by an azole drug. Fluconazole as a single drug was the most frequent treatment option. The optimal treatment strategy for uncomplicated coccidioidomycosis in individuals without risk factors for severe or disseminated disease remains uncertain. Even though the use of amphotericin B followed by fluconazole or itraconazole is recommended based on the guidelines from the Ministry of Health in Brazil, other authors suggest that the use of amphotericin depends on the form of disease presentation^24^ ^,^ ^25^. The Infectious Disease Society of America (IDSA) recommends initiating antifungal treatment with orally absorbed azole antifungals (e.g., fluconazole) for patients who, at the time of diagnosis, present with significantly debilitating illness^26^. According to IDSA, amphotericin B should be reserved for cases where there is contraindication for azole use^26^. Owing to its low cost, excellent oral absorption ( >90%), few adverse effects, and good tolerability and availability in both oral and intravenous formulations, fluconazole is one of the most frequently prescribed drugs for coccidioidomycosis treatment^27^. 

In this study, 83.8% of the patients were cured of the disease, with only one death registered. The short interval of time between the onset of symptoms and the first medical evaluation at the São José Hospital (20 days), along with the fact that the patients presented with acute disease and were properly treated, could explain the favorable outcome. Data show that 60% of patients with *Coccidioides* infection remain asymptomatic. Although the disease progresses into acute pulmonary coccidioidomycosis in the remaining patients, the outcomes are usually favorable^2^. In a large epidemiological survey on coccidioidomycosis in the United Sates, a progressive reduction in mortality from the disease was observed among hospitalized patients. This reduction was possibly related to early diagnosis and proper infection management^28^. Therefore, acute coccidioidomycosis may have a favorable prognosis, especially when diagnosed early and treated properly. 

Coccidioidomycosis was more prevalent in the central regions of the State of Ceará. Knowledge of the local epidemiology and clinical manifestations of coccidioidomycosis is important for a better understanding of the disease. The diagnosis of coccidioidomycosis requires a high index of suspicion. Therefore, careful evaluation of the clinical and epidemiological conditions of the patient, in addition to pulmonary radiological findings, will help raise early suspicion of the disease, and may possibly lead to an early diagnosis. 
